# Trends in Cancer Mortality Disparities Between Black and White Individuals in the US, 2000-2020

**DOI:** 10.1001/jamahealthforum.2023.4617

**Published:** 2024-01-12

**Authors:** Anjali Gupta, Tomi Akinyemiju

**Affiliations:** 1 Department of Population Health Sciences, Duke University School of Medicine, Durham, North Carolina; 2 Stanford University School of Medicine, Stanford, California; 3 Duke Cancer Institute, Duke University School of Medicine, Durham, North Carolina

## Abstract

This cross-sectional study compares trends in estimated age-adjusted cancer mortality rates between non-Hispanic Black and non-Hispanic White individuals in the US from 2000 to 2020.

## Introduction

Cancer is the second leading cause of mortality in the US, with over 600 000 cancer deaths projected to occur in 2023.^1^ The American Cancer Society estimates that cancer mortality has declined by 33% since 1991,^1^ with improvements in cancer prevention, screening, diagnosis, and treatment. However, racial and ethnic disparities remain, with non-Hispanic Black (hereafter, Black) patients often faring worse than non-Hispanic White (hereafter, White) patients.^2^ This study builds on prior work by Lawrence et al^3^ by describing racial disparities in cancer mortality between Black and White US individuals in the past 2 decades.

## Methods

This cross-sectional study used publicly available deidentified data from the National Center for Health Statistics^4^; thus, Duke University waived approval and informed consent. We followed the STROBE guideline.

SEER*Stat software, version 8.4.1, was used to estimate age-adjusted cancer mortality rates among Black and White individuals for each year between January 2000 and December 2020. Mortality rates were age-standardized to the 2000 US population. We evaluated mortality for all cancers combined and separately for the 4 most common cancer types^1^—female breast, prostate, lung and bronchus, and colorectal—and stratified by sex. We assessed cancer mortality rate ratios and absolute differences between Black and White individuals for each year. Using the Joinpoint Regression Program, version 5.0.2, average annual percentage changes (AAPCs) in mortality were calculated. Joinpoint regression comparability testing for parallelism in annual percentage change was conducted to compare trends over the study period by race and ethnicity. Two-sided *P* < .05 was significant.

## Results

**Figure 1.**
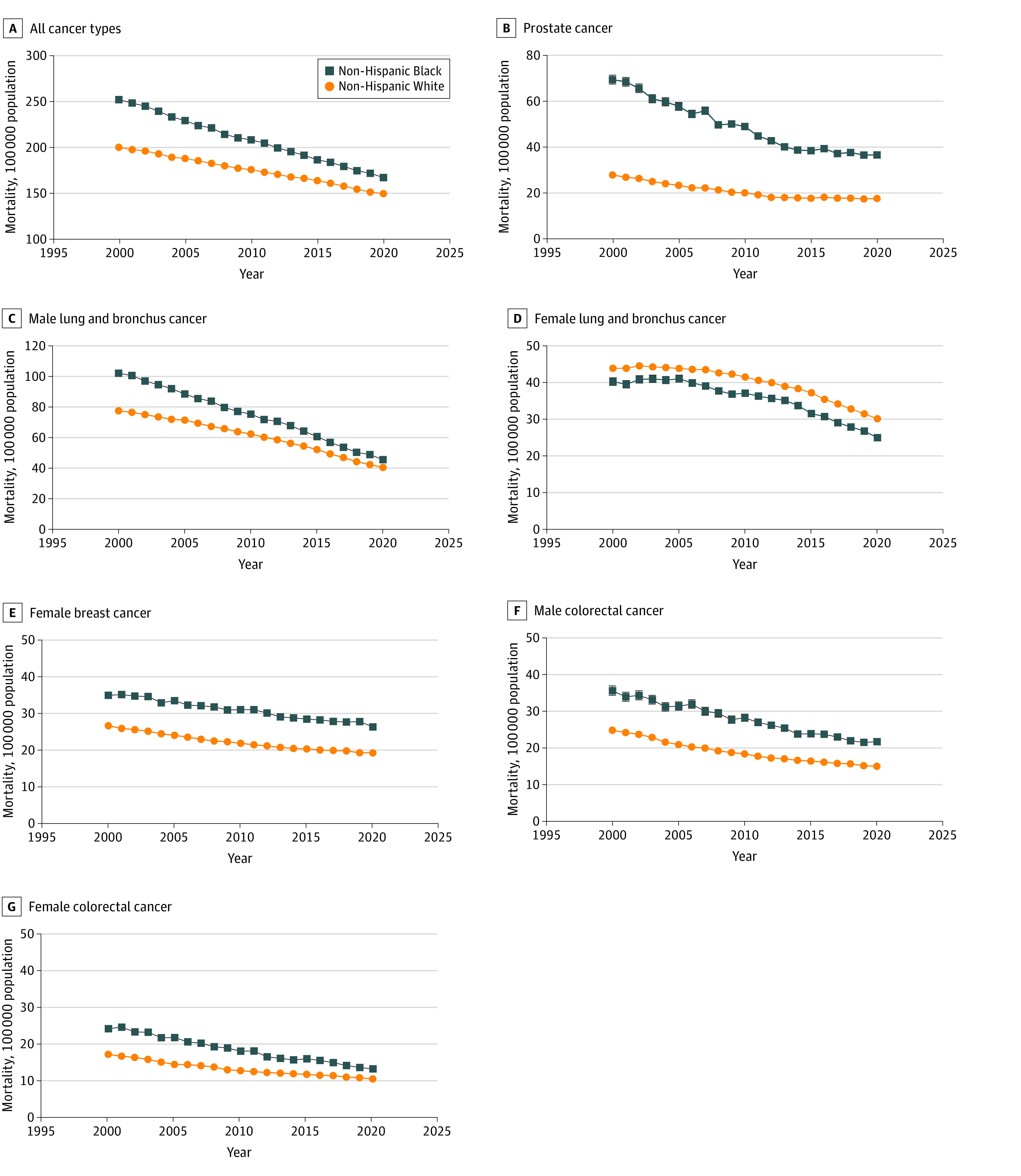
Age-Adjusted Cancer Mortality Rates Whiskers indicate 95% CIs; in some cases, 95% CIs are too narrow to be visible.

Age-adjusted mortality rates are presented in Figure 1 by race and ethnicity, sex, and cancer type. In 2000, the rate was 251.7 per 100 000 population among Black individuals and 199.7 per 100 000 population among White individuals, decreasing to 166.8 per 100 000 population (AAPC, −2.04% [95% CI, –2.07% to –2.00%]) and 149.3 per 100 000 population (AAPC, −1.44% [95% CI, –1.48% to –1.39%]), respectively, by 2020 (*P* < .001 for trend). Between 2000 and 2020, declines in cancer mortality were observed for each cancer type for both groups. However, Black individuals consistently experienced higher mortality than White individuals for all cancers except female lung and bronchus.

**Figure 2.**
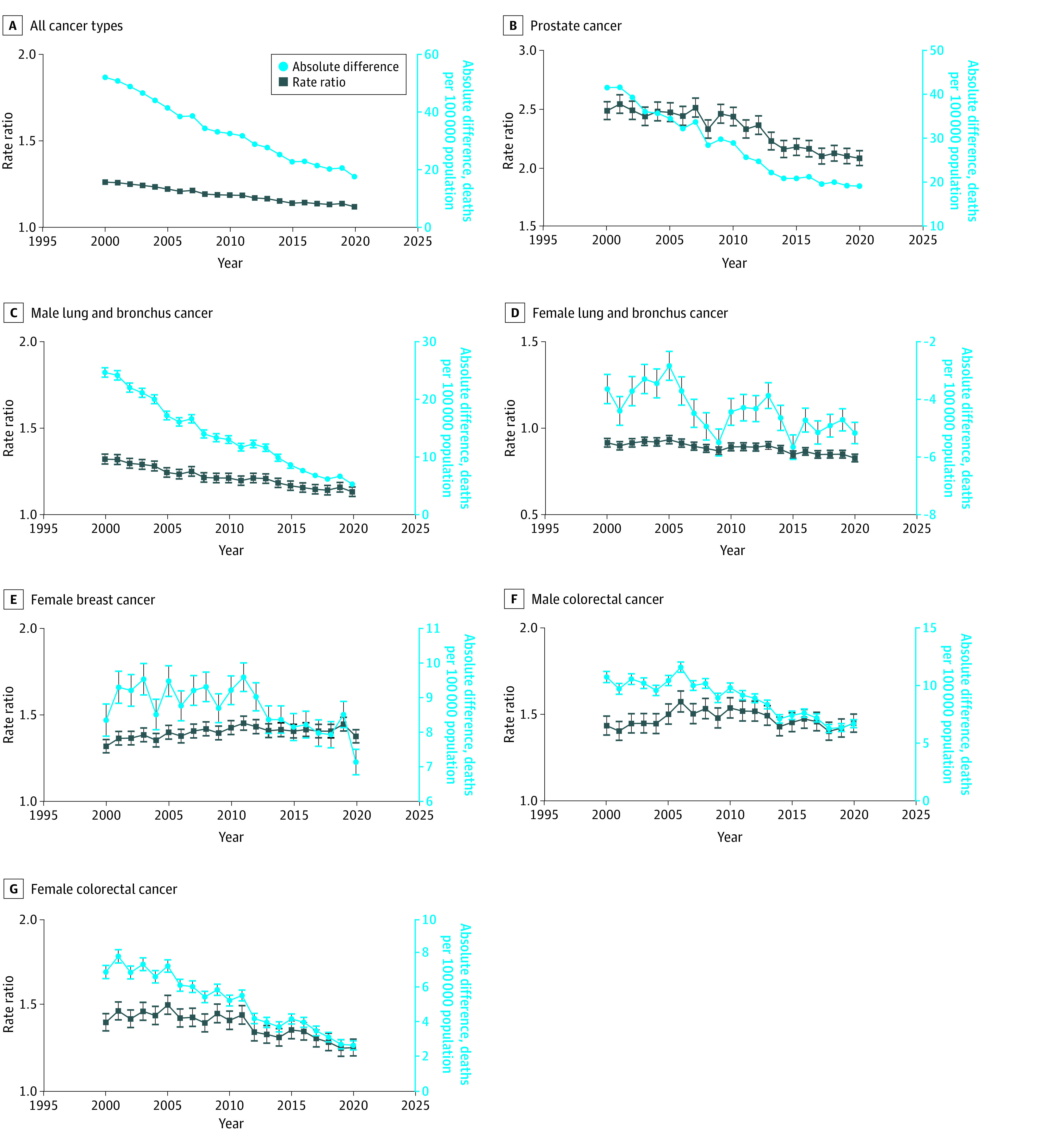
Cancer Mortality Rate Ratios and Absolute Rate Differences for Non-Hispanic Black Individuals and Non-Hispanic White Individuals Whiskers indicate 95% CIs; in some cases, 95% CIs are too narrow to be visible. The reference group for all comparisons by race and ethnicity is non-Hispanic White individuals.

The overall cancer mortality rate ratio between Black and White individuals decreased from 1.26 (95% CI, 1.25-1.27) in 2000 to 1.12 (95% CI, 1.11-1.13) in 2020, with a corresponding decrease in absolute rate differences (51.99 to 17.54 per 100 000 population) (Figure 2). However, the rate ratio increased for female breast cancer (1.31 [95% CI, 1.27-1.35] to 1.37 [95% CI, 1.33-1.41]) and male colorectal cancer (1.44 [95% CI, 1.38-1.49] to 1.45 [95% CI, 1.40-1.50]), and considerable disparities remained for prostate (2.49 [95% CI, 2.41-2.56] to 2.08 [95% CI, 2.02-2.14]), male lung and bronchus (1.32 [95% CI, 1.29-1.35] to 1.13 [95% CI, 1.10-1.16]), and female colorectal (1.40 [95% CI, 1.36-1.46] to 1.26 [95% CI, 1.21-1.31]) cancer across the study period.

## Discussion

Although US age-adjusted cancer mortality rates declined significantly between 2000 and 2020, substantial racial and ethnic disparities persisted for many common and preventable cancers, including female breast and male colorectal cancer. Cancer disparities arise from a confluence of factors, including structural racism, medical mistrust, health care access inequities, poor socioenvironmental conditions, aggressive tumor biology, and genetic ancestry.^2,5,6^ Multilevel strategies to strengthen health system relationships with minority communities, improve access to timely health care, investigate racial and ethnic differences in tumor biology, and increase enrollment of minority groups in clinical trials will be crucial to bridging mortality disparities. The main limitation was possible misclassification of race and ethnicity and cause of death. Our results underscore the importance of sustained, focused efforts to reduce cancer burden among Black patients across the continuum of cancer care.

## Notes

Supplement. Data Sharing Statement
