# Supplementary material for: Trends in Cancer Mortality Disparities Between Black and White Individuals in the US, 2000-2020
Source: JAMA Health Forum. 2024 Jan 12;5(1):e234617. doi: 10.1001/jamahealthforum.2023.4617 (PMC10787320; doi:10.1001/jamahealthforum.2023.4617)
Supplement: Supplement. — Data Sharing Statement [file jamahealthforum-e234617-s001.pdf]

Data Sharing Statement

Gupta. Trends in Cancer Mortality Disparities Between Black and White Individuals in the US, 2000-2020. *JAMA Health Forum*. Published January 12, 2024.
doi:10.1001/jamahealthforum.2023.4617

Data
Data available: No

Additional Information
Explanation for why data not available: This analysis utilized secondary data from the SEER Program and NCHS. More information and instructions to request these data are available here: https://seer.cancer.gov/data/access.html.
